# HEalth Data Research OMOP Network (HERON‐UK), A Real‐World Evidence Federated Network of Hospital and Primary Care Electronic Health Records Including 57 Million Individuals and 7 Data Partners in the United Kingdom

**DOI:** 10.1002/pds.70526

**Published:** 2026-11-13

**Authors:** Cecilia Campanile, Alex E. Knight, Hiba Junaid, Jennifer C. E. Lane, Usama Rahman, Ben Eaton, Xavier L. Griffin, Andy South, Ana Cavalcante, Stefan Piatek, Baptiste Briot Ribeyre, Steve Harris, Timothy Howcroft, Vishnu Chandrabalan, Peter S. Hall, Mahéva Vallet, Marta Pasikowska, Nicola Symmers, Colin McLean, Spyro Nita, Daniel Dedman, Zara Cuccu, Stelios Theophanous, Geoff Hall, Edward Bolton, Shiren Patel, Daniel Key, Lydia Briggs, Prasiddha Khadka, David Seymour, Elin Rowlands, Danielle Newby, Daniel Prieto‐Alhambra, Edward Burn, Martí Català

**Affiliations:** ^1^ Health Data Sciences, Nuffield Department of Orthopaedics, Rheumatology and Musculoskeletal Sciences (NDORMS) University of Oxford Oxford UK; ^2^ Health Data Research UK (HDR UK) London UK; ^3^ Barts Bone and Joint Health Queen Mary University of London London UK; ^4^ Barts Health NHS Trust, NHS Trust London UK; ^5^ Barts Life Sciences London UK; ^6^ University College London Hospitals Biomedical Research Centre London UK; ^7^ Institute of Health Informatics University College London London UK; ^8^ Lancashire Teaching Hospitals NHS Foundation Trust Preston UK; ^9^ DataLoch, Edinburgh Cancer Informatics University of Edinburgh Edinburgh UK; ^10^ Clinical Practice Research Datalink (CPRD) Medicines and Healthcare Products Regulatory Agency (MHRA) London UK; ^11^ Leeds Teaching Hospitals NHS Trust London UK; ^12^ Data Research, Innovation and Virtual Environments (DRIVE) Great Ormond Street Hospital for Children London UK; ^13^ Department of Medical Informatics Erasmus MC University Rotterdam the Netherlands

## Abstract

**Introduction:**

The increasing availability of routinely collected healthcare data presents opportunities to generate real‐world evidence (RWE) to inform regulatory, clinical, and policy decisions. However, fragmented data sources, heterogeneous data structures, and inconsistent coding systems hinder reliable, large‐scale analyses. Federated data networks adopting a common data model, such as the Observational Medical Outcomes Partnership (OMOP) Common Data Model (CDM), offer a standardised, privacy‐preserving approach to addressing these challenges.

**Methods:**

We established a federated data network comprising seven UK‐based partners, each maintaining routinely collected healthcare data mapped to the OMOP CDM. A central coordinating centre develops and distributes standardised analytical code for local execution within each partner's secure environment. Only aggregated, non‐identifiable results are returned to the coordinating centre, enabling reproducible analyses while preserving data privacy. Partner c followed a three‐stage process comprising database description, benchmarking, and comprehensive data characterisation to assess data quality, support study feasibility, and improve understanding of the available data.

**Results:**

The network comprises five English National Health Service (NHS) trusts, one primary care data provider, and one Scottish NHS trust. Collectively, the participating databases contain records representing approximately 57.2 million individuals, 1.73 billion drug exposures, and 854 million condition records. The population estimate does not account for potential overlap between data sources.

**Conclusions:**

We established a large‐scale UK federated healthcare data network using the OMOP CDM, integrating diverse primary and secondary care data sources within a privacy‐preserving analytical framework. The network provides an infrastructure for standardised, reproducible, multi‐database observational research and supports the generation of RWE to inform healthcare decision‐making.

## Key Points

- Federated OMOP‐Based Network: HERON‐UK executes shared analysis code across seven UK data partners mapped to OMOP and returns only aggregated outputs; no patient‐level data leave data partner environments.
- Structured Onboarding: All partners completed a standardised onboarding process to ensure technical compatibility and data readiness.
- Large‐Scale Coverage: The network includes data on 57.2 million individuals, 1.73 billion drug exposures, and 854 million conditions records.
- Feasibility demonstrated: Two completed studies have shown the network can produce robust real‐world evidence in practice.

## Plain Language Summary

Healthcare systems collect a huge amount of patient information during routine care. This data can help improve medical decisions and public health policy, but it's often stored in different formats across hospitals and regions, making it hard to use in research. To solve this, we created a UK‐wide network of seven healthcare partners who agreed to use the same structure to organise their data. This structure is known as the Observational Medical Outcomes Partnership Common Data Model, which helps standardise data without sharing any personal details. Each partner keeps full control of their data, running the same research code locally and only sharing summary results. This approach protects privacy while allowing researchers to work together more effectively. Each partner provided a description of the data and ran some code to check its quality and what it contains. Our network includes hospitals and GP data from across England and Scotland, covering around 57 million people. Together, the data include over 1.7 billion records of medicines prescribed and over 850 million records of health conditions, providing a powerful resource for health research that is both secure and consistent.

## 1 Introduction

The UK's National Health Service (NHS) captures a vast amount of real‐world data across numerous providers [1, 2]. Performing large‐scale research using real‐world data from multiple providers operating across the four UK nations can be facilitated by mapping and transforming the data to a Common Data Model (CDM), such as the Observational Medical Outcomes Partnership (OMOP) CDM [3, 4].

The OMOP CDM is a person‐centric CDM that standardises the structure, vocabulary, and relationships of clinical data, enabling harmonised analyses across heterogeneous sources. This can make data more discoverable, analysis more efficient, and enable its federation across multiple sites. Its widespread international adoption has also led to the development of an extensive ecosystem of open‐source tools for data curation and analysis, making OMOP an attractive option for both data partners and study coordinators. OMOP has become one of the most widely adopted CDMs for observational health research in the UK, and NHS England identifies OMOP as the preferred standard for research‐related use cases [5, 6]. Its adoption has been increasing, thanks to initiatives such as the European Health Data & Evidence Network (EHDEN) project [7], which supported 22 UK data partners, such as NHS hospitals, universities, and registries, to transform their data. However, use of these assets for research or other applications has been more limited.

The HEalth data Research OMOP Network (HERON‐UK) [8] is an OMOP‐based network established by Health Data Research UK (HDR UK) to enable efficient, cross‐nation real‐world evidence (RWE) studies across the UK. Built on transformed data from the EHDEN project and other sources, HERON‐UK aims to demonstrate the value of the OMOP CDM as an enabler of efficient cross‐nation research studies within the UK, and to transition to a more sustainable “pull” model—where data partners are paid for the *use* of their data—rather than a “push” model where they are paid to *convert* data to the OMOP CDM. This approach has been demonstrated successful, for example by the DARWIN‐EU project [9], where the European Medicines Agency funds studies to answer regulatory questions using Real‐World Data.

## 2 Network Set‐Up and Data Partner Evaluation

HERON‐UK was initially funded as a pilot programme, through two open calls. The first call [10] was to recruit an expert team to act as the network coordination centre. The Health Data Sciences research group at the University of Oxford was selected as the HERON‐UK coordination centre, with responsibility for leading studies, onboarding data partners, providing common tools and protocols, and coordinating network operations. The second call [11] aimed to recruit up to seven data partners who would contribute their data and expertise to the network. Proposals from data partners were scored and ranked by a review panel consisting of stakeholders including regulatory, public health, and Health Technology Assessment (HTA) national agencies, a Patient and Public Involvement and Engagement (PPIE) representative, and members of the coordinating centre and HDR UK.

The selected data partners underwent an onboarding process to join the network that comprised three stages:

1. *Description of the database:* to get insight into the information mapped in each database and their source data.
2. *Benchmarking:* data partners had to demonstrate their ability to run 'R' code on their OMOP CDM instance and demonstrate the feasibility of subsequent steps.
3. *Characterisation:* data partners ran code that returned more detailed information about their data to inform study feasibility.

## 3 Description of Data Partners

HERON‐UK includes seven data partners, each contributing data mapped to the OMOP CDM, and representing a wide range of healthcare settings across the UK. Together, they provide access to diverse real‐world data sources across the UK (Figure 1), enabling large‐scale observational research. Data partner specific geographic coverage is presented in Figures S1–S7. In its current form, the network offers satisfactory coverage of the populations of England and South East Scotland but under‐represents Wales and Northern Ireland. Expanding to include data sources from these nations is a priority to achieve the best possible representation of the UK population.

**FIGURE 1:**
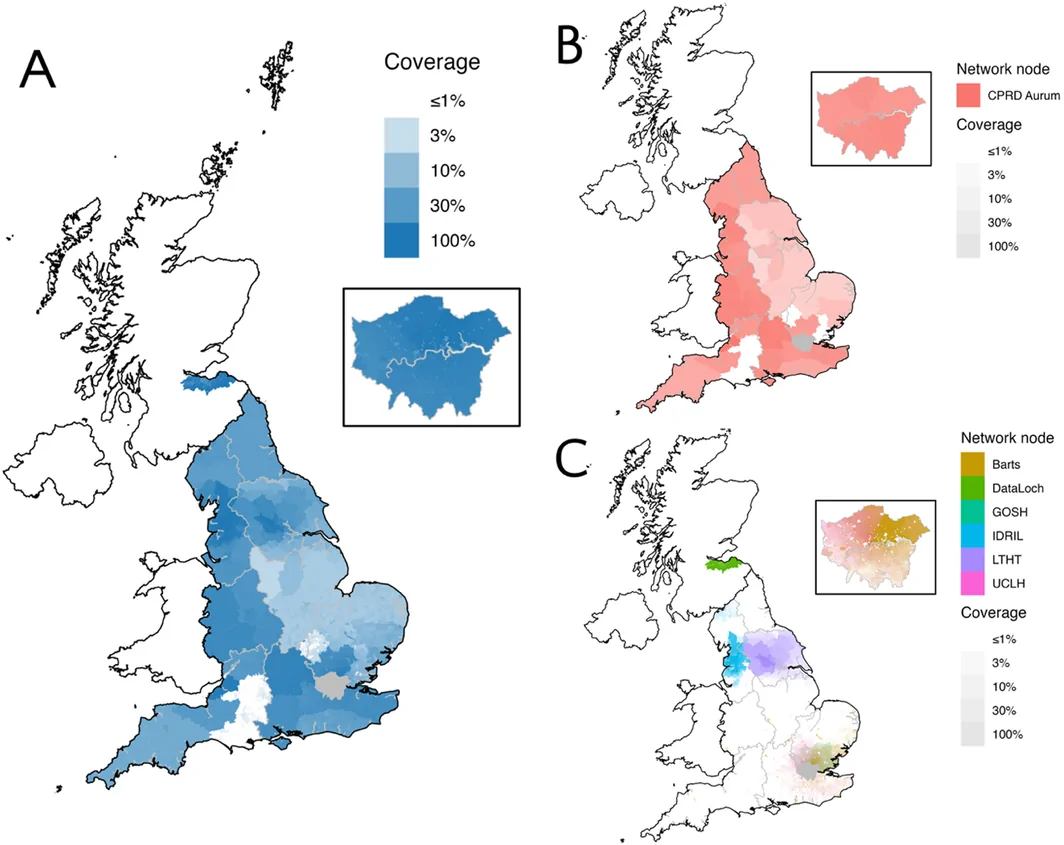
Coverage of the HERON‐UK network, shown on a map of the UK with a close‐up inset of London (A) Overall network. (B) Only primary care data partners. (C) Only secondary care data partners. Barts, Barts Health NHS Trusts; UCLH, University College London Hospitals NHS Trust; LTHT, Leeds Teaching Hospitals NHS Trust; IDRIL, Lancashire Teaching Hospitals; GOSH, Great Ormond Street Hospital NHS Foundation Trust; CPRD Aurum, Clinical Practice Research Datalink Aurum.

For the description phase data partners had to provide:

1. A description of their database.
2. OMOP CDM details: CDM version, OMOP vocabulary version used to map the data, source vocabularies and observation period definition.
3. Coverage across the United Kingdom. Person counts at post code district level (LTHT), Lower layer Super Output Areas level [12] (Barts, IDRIL, UCLH), Data Zone level [13] (DataLoch) or Integrated Care Board level [14] (CPRD Aurum, GOSH) were provided to assess geographic coverage.
4. Governance: the internal procedure to validate the aggregated data and authorise its release, including required checks and any protocol submissions.

Tables S1–S7 provide the onboarding description details of each data partner. A brief description of the seven data partners can be found below:

Clinical Practice Research Datalink Aurum (CPRD Aurum) [15] is a primary care electronic health records (EHR) database collected from general practitioner (GP) practices in England using the EMIS management software system. It includes approximately 49 million patients and captures anonymised data on demographics, clinical events, investigations, referrals, and prescriptions. The database provides broad and longitudinal coverage of the English population and is widely used in observational research.

Great Ormond Street Hospital NHS Foundation Trust (GOSH) [16] is a specialist tertiary care paediatric hospital in the UK that receives children with complex and/or rare conditions. Many patients require long‐term care and follow‐up, resulting in highly detailed clinical data. The database includes over 200 000 patients with inpatient or outpatient encounters between April 2019 and February 2025.

Leeds Teaching Hospitals NHS Trust (LTHT) [17] contributes data on approximately 1.5 million patients with diagnoses or admissions since 2003. The dataset receives data from multiple hospital systems and includes detailed information related to cancer care, including diagnosis, treatment, staging, biomarkers, and clinical trial participation.

Lancashire Teaching Hospitals (IDRIL) [18] dataset provides hospital‐based data from the Northwest of England, covering approximately 1.5 million patients. The dataset includes a wide range of clinical information, such as outpatient clinic attendances, laboratory test results (including blood tests and microbiology), and detailed cancer pathway data, including staging and grading.

University College London Hospitals NHS Trust (UCLH) [19] is one of the largest NHS trusts in the UK, providing first‐class acute and specialist services across six hospitals in central London. It contributes a dataset of 6 million patients. The UCLH patient population includes national referrals for tertiary and quaternary services (cancer, neurology etc.) and general medical admissions from an inner‐city teaching hospital that treats > 1 million outpatients per year and has > 100 000 inpatient admissions.

Barts Health NHS Trust (Barts) [20] is the largest NHS hospital group in the UK. It contributes a dataset of approximately 3 million patients from across its six hospitals in East London: The Royal London Hospital, Newham General Hospital, Whipps Cross Hospital, Mile End Hospital, St Bartholomew's Hospital, and the London NHS Nightingale Hospital, a purpose‐built COVID‐19 hospital. The dataset spans from 2012, with comprehensive and consistent records available from 2015/2016 onwards.

DataLoch's [21, 22] OMOP mapped data includes records from approximately 1.7 M individuals in the geographical area of South East Scotland. It incorporates Lothian extracts from four national data sources: PHS Scottish Morbidity Records SMR00 (outpatient), SMR01 (inpatient), and SMR06 (cancer registry), and the PIS dataset for community prescribing information. These patients have been linked to historical information for hospitalisations, drug prescriptions, laboratory results, SIMD quintiles, GP‐recorded BMI, alcohol consumption and smoking status, and deaths data from the National Records of Scotland.

## 4 Operational Test

HERON‐UK operates under a federated analytics framework (Figure 2) which facilitates collaborative research across distributed data sources while preserving the confidentiality of patient‐level information [23]. In this setup, data partners retain full control over their individual‐level data and are responsible for executing analytical code locally and returning results to the coordinating centre. Data partners can apply their own ethics rules, such as small‐number suppression and airlock processes to the data. The coordinating centre does not have access to raw data but is tasked with developing and distributing standardised R‐based analysis scripts to all participating partners.

**FIGURE 2:**
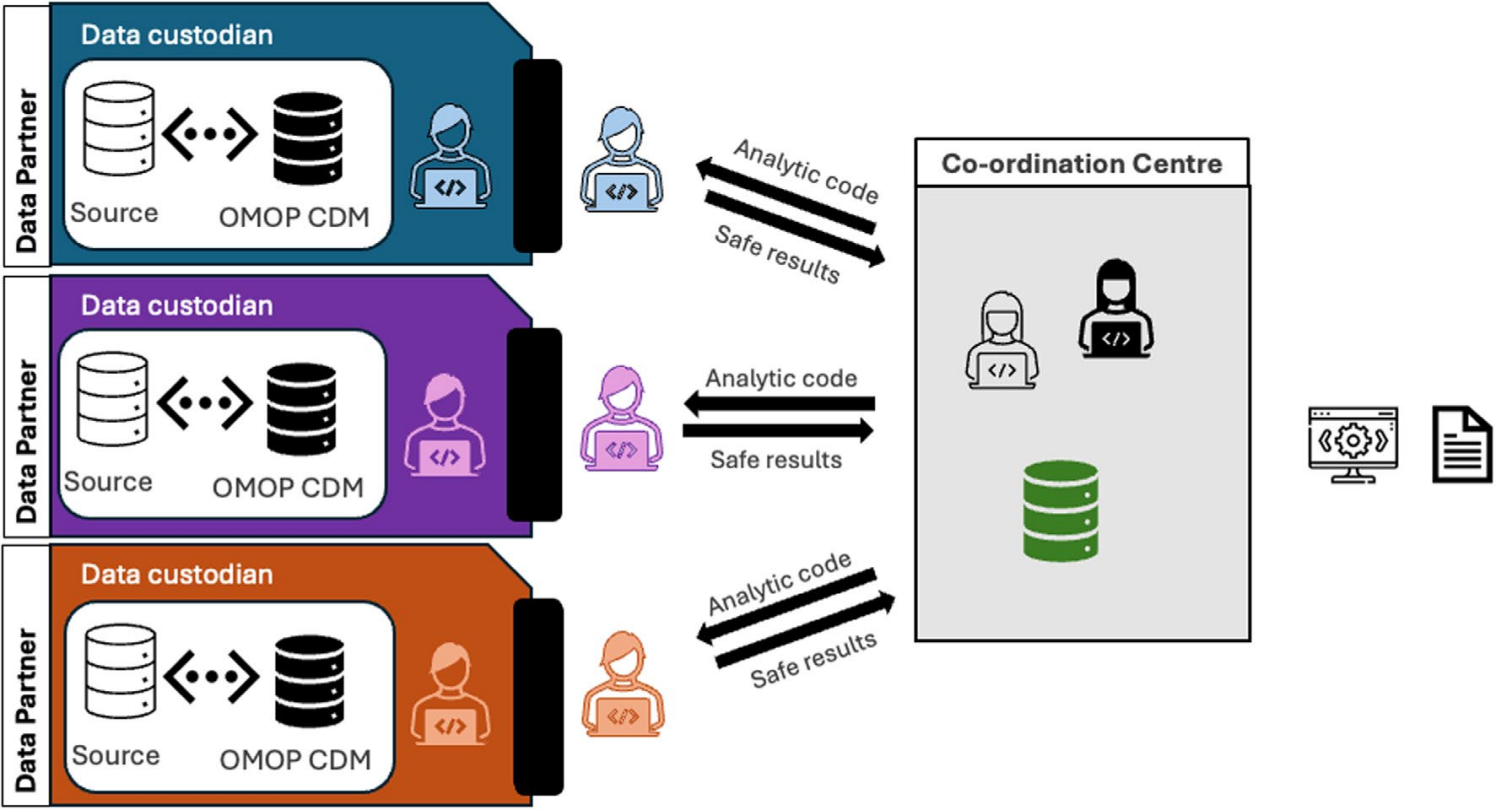
Federated analytics diagram. Data partners map their source data to the OMOP CDM, with both datasets remaining securely within each partner's environment. The coordinating centre supplies analytic code, which is executed locally against the OMOP CDM instance. Only aggregated, non‐identifiable results are returned to the coordinating centre for central analysis, making the outputs safe to share while preserving patients' privacy.

Analysis outputs are restricted to aggregated results only in a standardised format exported to CSV (comma separated value) files. To meet each site's information governance requirements, any aggregated cell count below the locally defined minimum threshold is suppressed. Aggregated outputs are returned to the coordinating centre via a secure file transfer mechanism.

All code and associated documentation are shared via the network's GitHub organisation: https://github.com/heron‐uk. GitHub is also used for issue tracking and version control across sites.

To verify that each site could run OMOP CDM federated analytics reliably, we designed a benchmarking project available at: https://github.com/heron‐uk/HERON‐UK‐benchmark. The benchmark aimed to confirm that core analytic packages could be successfully executed within the local environments of each data partner. Specifically, the benchmark tested functionality and performance for several R packages designed for OMOP CDM analytics that are used for studies in the network: CDMConnector [24], CohortConstructor [25], IncidencePrevalence [26], CohortCharacteristics [27], DrugUtilisation [28], and CodelistGenerator [29].

The benchmarking code recorded the elapsed time required to complete several representative tasks, such as counting rows in key tables and instantiating acetaminophen and warfarin cohorts. Results from each site were submitted to the coordination centre and visualised in a Shiny dashboard: https://dpa‐pde‐oxford.shinyapps.io/benchmark‐hdruk/. This allowed the coordination team to check that the execution times were consistent with expected data sizes. Running the code without any errors also ensured that OMOP CDM tables were populated and correctly formatted. Feedback was provided whenever results failed to meet standards; where the benchmark identified database‐side problems, the Data Partner corrected them and reran the code to confirm resolution.

## 5 Characterisation

To support study planning and assess the suitability of datasets within HERON‐UK, a comprehensive database characterisation process was conducted across all data partners as a final part of their onboarding. This effort aimed to establish a shared understanding of the structure, content, coverage, and quality of the available data, and to identify potential limitations that may impact future analyses.

The study code generated the following summaries:

– *Metadata:* Key metadata elements were extracted from each data source, including the number of individuals, the data coverage period, and vocabulary versions. Keeping track of the different vocabulary versions is important as new concepts are added at every vocabulary release (twice a year), potentially affecting cohort identification and cross‐site comparability. Data Partners are expected to keep mappings updated to the latest vocabulary to align versions across the network.
– *Observation period:* The OMOP CDM's ‘observation_period’ table defines the time span during which each individual is in observation (i.e., when the data source is expected to have complete and reliable records for that person). Metrics were calculated, such as the number of individuals under observation per year, the number of observation periods per person, and the duration of these periods.
– *Population characteristics:* Demographic profiles were summarised for individuals in observation to describe the populations represented within each data source and identify differences in population coverage across data partners.
– *Clinical tables:* Clinical tables were assessed for data completeness and conformance with the OMOP CDM. Checks included the completeness of key fields, the appropriate use of standard and source concepts and their expected domains, vocabulary usage, record counts per person, and annual trends in record availability. These assessments were used to identify potential missingness, mapping issues and unexpected patterns in the content or temporal coverage of the data.
– *Concept counts:* The frequency of each concept ID within the clinical tables such as diagnoses, procedures and drug exposures.

The code is primarily based on the OmopSketch [30] R package. Analyses were restricted to data from 2012 onward. All results were stratified by sex and by age group (0–19, 20–39, 40–59, 60–79, 80+) to enable consistent comparisons across datasets. The code is publicly available on GitHub: https://github.com/heron‐uk/HDRUK‐Characterisation.

Results were compiled and visualised in a shiny app: https://dpa‐pde‐oxford.shinyapps.io/HDRUK‐Characterisation/. Concept ID counts were not included in the public dashboard as they are meant to be used only for the internal feasibility of the different studies. The network currently includes data on approximately 57.2 million individuals (Table 1), although this figure does not incorporate any adjustments for duplicate person‐level entries. As the contributing data sources are not linkable at the individual level, each dataset effectively represents an independent population sample.

**TABLE 1:** Database characterisation showing the number of subjects, observation period range, database management system, OMOP CDM version and OMOP vocabulary version for each one of the HERON‐UK data partners.

	Barts	UCLH	LTHT	IDRIL	DataLoch	CPRD Aurum	GOSH
Number of subjects	3 235 843	1 129 661	1 486 578	1 533 222	1 763 406	49 359 991	204 159
Observation period range	2012–2025	2019–2025	2003–2025	2005–2025	1984–2025	1995–2024	2019–2025
Database management System	SQL server	PostgreSQL	SQL server	SQL server	SQL server	SQL server	SQL server
CDM version	5.4	5.3	5.4	5.4	5.4	5.4	5.4
Vocabulary version used	v5.0 30‐AUG‐24	v5.0 27‐FEB‐25	v5.0 30‐AUG‐24	v5.0 30‐AUG‐24	v5.0 27‐FEB‐25	v5.0 30‐AUG‐24	v5.0 30‐AUG‐24

Abbreviations: Barts, Barts Health NHS Trusts; GOSH, Great Ormond Street Hospital NHS Foundation Trust; IDRIL, Lancashire Teaching Hospitals; LTHT, Leeds Teaching Hospitals NHS Trust; UCLH, University College London Hospitals NHS Trust.

The age and sex distribution of the individuals in observation varied across data partners, reflecting different care settings and populations. GOSH, as a specialist paediatric provider, had the youngest population in the network, with a median age of six (IQR 0–12) years (Table 2). DataLoch, Barts, and CPRD Aurum had slightly younger populations, with median ages between 31 and 33 years compared to UCLH, IDRIL and LTHT which had slightly older cohorts, with median ages of 38, 43 and 50 years, respectively. Across all data partners, there were slightly more females than males, with the proportion of females ranging from 50.2% to 54.6%, consistent with the national estimate of 51% [31]. Furthermore, age distributions across the different data partners showed Barts, CPRD AURUM and DataLoch had similar population age distribution, whereas IDRIL and UCLH showed a skew to older populations with LTHT showing the highest representation of the oldest populations with GOSH again showing the youngest age distribution (Figure 3).

**TABLE 2:** Characteristics of individuals in observation between 2012 and data extraction. Index date to calculate age is defined as the later of entering the database and 1st of January 2012.

	Barts	UCLH	LTHT	IDRIL	DataLoch	CPRD Aurum	GOSH
Number of individuals	2 908 703	995 469	945 056	1 270 144	1 627 000	33 954 151	204 159
Female %	51.01%	54.65%	52.59%	52.12%	52.23%	50.84%	50.23%
Age median [Q25—Q75]	31 [20–47]	38 [26–57]	50 [28–69]	43 [24–62]	33 [19–53]	31 [18–49]	6 [0–12]

Abbreviations: Barts, Barts Health NHS Trusts; GOSH, Great Ormond Street Hospital NHS Foundation Trust; IDRIL, Lancashire Teaching Hospitals; LTHT, Leeds Teaching Hospitals NHS Trust; UCLH, University College London Hospitals NHS Trust.

**FIGURE 3:**
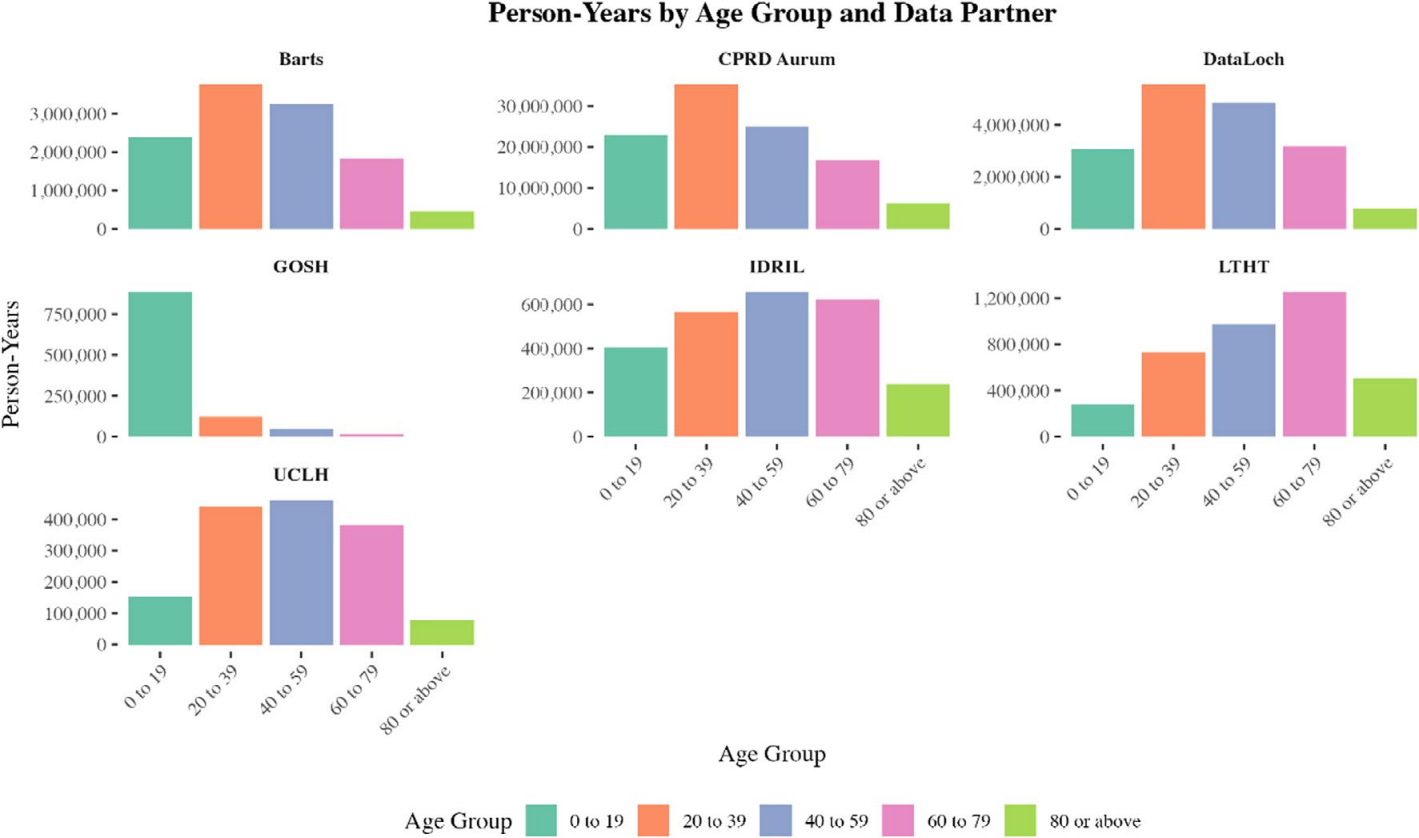
Number of person‐years in observation stratified by age group and data partner. Barts, Barts Health NHS Trusts; CPRD Aurum, Clinical Practice Research Datalink Aurum; UCLH, University College London Hospitals NHS Trust; LTHT, Leeds Teaching Hospitals NHS Trust; IDRIL, Lancashire Teaching Hospitals; GOSH, Great Ormond Street Hospital NHS Foundation Trust.

A total of 1.73 billion drug exposure records and 854 million condition occurrence records were recorded across the Network (Table 3). CPRD Aurum is the biggest contributor in terms of records (~90% of the records of the network) as it is a primary care dataset with more follow‐up and a larger denominator.

**TABLE 3:** Number of records in observation (in millions) and distinct concepts (identifiers that represent a standardised clinical entity, e.g., a drug, a diagnostic, a procedure, etc.) per data partner and clinical domain.

	Barts	UCLH	LTHT	IDRIL	DataLoch	CPRD Aurum	GOSH
*Drugs*							
Records	36.9	31.8	3.0	20.2	158.6	1608.9	7.8
Concepts	1447	3386	576	736	6445	11 392	3858
*Conditions*							
Records	26.0	6.3	5.0	14.4	6.8	801.8	8.5
Concepts	19 859	14 909	4371	6342	4565	32 036	4051
*Procedures*							
Records	9.7	24.9	1.6	8.4	2.3	1285.2	1.8
Concepts	8383	2769	3447	4354	3787	15 742	2295
*Device*							
Records	1.5	4.9	0	0.9	0	155.4	0
Concepts	15	236	—	17	—	31 838	—
*Measurements*							
Records	730.7	539.6	2.2	340.9	345.1	4368.7	115.4
Concepts	180	921	161	359	96	7377	25

Abbreviations: Barts, Barts Health NHS Trusts; CPRD Aurum, Clinical Practice Research Datalink Aurum; GOSH, Great Ormond Street Hospital NHS Foundation Trust; IDRIL, Lancashire Teaching Hospitals; LTHT, Leeds Teaching Hospitals NHS Trust; UCLH, University College London Hospitals NHS Trust.

The characterisation of the observation periods for each data partner highlighted differences in how observation periods were being defined across data partners (as described in Tables S1–S7). Most datasets included a single observation period per person, while in IDRIL, some individuals had up to seven observation periods since 2012. The median duration of observation also varied, reflecting differences in extraction approaches and ETL configurations. These inconsistencies highlighted the need to standardise observation period definitions across the network to ensure more comparable and reliable analyses in future studies.

## 6 Network Activities

The first year of the HERON‐UK network was a pilot phase, with the aim of demonstrating the feasibility and the value of an OMOP‐based federated network including primary care, hospital, and linked UK datasets. To this end, in addition to the recruitment and onboarding of the data partners, we carried out two research studies using the network's data.

The first study was titled “The use of antibiotics with high resistance potential in primary and secondary care in the United Kingdom” [32] and involved all the data partners. A second study, “Utilisation of high‐cost medicines in hospital settings in the United Kingdom” [33] used a subset of the data partners with secondary care data. These studies demonstrate how the diverse data sources in the network, together with the detailed characterisation of their datasets, enable a wide range of study topics and designs. The results of these studies will be published separately.

A “Study‐a‐thon” [34] was held which brought together representatives of all data partners for an intensive week of activity. The programme included the completion of onboarding and the execution of the research studies, as well as reaching agreement on technical topics such as the definition of observation periods. The results were also presented to the stakeholder review panel.

## 7 Network Limitations

CPRD Aurum is a large primary care database covering practices in England, and some individuals included in CPRD Aurum are therefore expected to overlap with those represented in other data sources within the network. Currently, individual‐level linkage between participating data sources is not available as the network is established within the federated analytics framework. The inability to link patients across data sources is inherent to the de‐identification process, as the identifiers required for linkage are not available within the network. Re‐identification of patients for the purpose of establishing such linkage is not permitted under the current legal framework. Consequently, the extent of this overlap cannot be identified or quantified. Network‐wide population counts should therefore not be interpreted as representing unique individuals, as the same individual may be represented twice appearing in primary and secondary care datasets. Overlap between secondary data sources should be minimal as patients in the UK have a reference hospital.

Although pooled estimates can be obtained, studies conducted within the network will report results separately for each database. Results will be presented alongside relevant database characteristics, including whether the data originate from primary or secondary care. While the other participating data sources include substantially fewer individuals than CPRD Aurum, they provide greater depth and granularity of secondary care information, including inpatient treatments, hospital‐administered medicines, clinical measurements, and procedures that may not be comprehensively captured in primary care data. These complementary characteristics broaden the range of research questions that can be addressed within the network.

## 8 Next Steps

The vision is that HERON‐UK becomes recognised as the natural destination for organisations with questions that require UK real‐world evidence to resolve. Those organisations can include regulators, academic and clinical researchers, and industries such as pharmaceuticals and medical devices. As a first step, in a proposed second year we plan to recruit additional data partners and to conduct new studies which further demonstrate the capabilities of the network. We will also refine the technical capabilities of the network and deliver training and support to our data partners. We will continue to co‐design the network and the studies it conducts with stakeholders including regulators, the public, and patients.

Areas of focus could include making the network more representative of the UK nations and incorporating new types of data sources such as NHS Secure Data Environments. As part of this effort, in year two we plan to onboard CPRD Gold to improve the network's coverage across UK nations, particularly Scotland, Wales, and Northern Ireland.

We aim to enrich data partners' OMOP assets, for example by enhancing the representation of procedure and device data, as we believe this will increase the breadth of studies the network can deliver. In parallel, identifying new sources of funding and partnerships with other initiatives will help ensure the network's sustainability and its ability to deliver further insights and real‐world evidence to improve health care outcomes. We expect that the learnings, software, network, and outputs from HERON‐UK will inform development of the UK's new Health Data Research Service [35].

## Funding

This work was supported by Health Data Research UK (2024.0225, 2024.0426, 2024.0427, 2024.0428, 2024.0429, 2024.0430, 2024.0431, 2024.0445), UK Research and Innovation, Department of Health and Social Care (England), NIHR Oxford Biomedical Research Centre.

## Conflicts of Interest

Prof. Daniel Prieto‐Alhambra is Principal Investigator for Real World Evidence studies sponsored by UCB Biopharma and Theramex, and a co‐investigator for a study sponsored by Eli Lilly, and previous co‐investigator for a study sponsored by Novartis. He has also been a co‐investigator in IMI‐funded projects (EHDEN, EU‐PEARL) and is currently involved in OPTIMA ONCOLOGY, while leading the IHI‐funded GREG (ihi‐greg.eu) programme. His research group has received public funding from NIHR, and the European Medicines Agency. Prof. Prieto‐Alhambra is a member of the ENCePP Steering Group.

## Supporting information


**Figure S1:** CPRD Aurum geographic coverage. Person counts are provided at Integrated Care Board level.
**Figure S2:** Barts Health NHS Trust (Barts) geographic coverage. Person counts are provided at Lower layer Super Output Areas level.
**Figure S3:** Great Ormond Street Hospital NHS Foundation Trust (GOSH) geographic coverage. Person counts are provided at Integrated Care Board level. Please note the colour legend of this data partner is different from the rest.
**Figure S4:** Lancashire Teaching Hospitals (IDRIL) geographic coverage. Person counts are provided at Lower layer Super Output Areas level.
**Figure S5:** Leeds Teaching Hospitals NHS Trust (LTHT) geographic coverage. Person counts are provided at post code district level.
**Figure S6:** DataLoch geographic coverage. Person counts are provided at Scottish Data Zone level.
**Figure S7:** University College London Hospitals geographic coverage. Person counts are provided at Lower layer Super Output Areas level.
**Table S1:** Great Ormond Street Hospital NHS Foundation Trust description details.
**Table S2:** Lancashire Teaching Hospitals description details.
**Table S3:** Leeds Teaching Hospitals NHS Trust description details.
**Table S4:** CPRD AURUM description details.
**Table S5:** Barts Health NHS Trust description details.
**Table S6:** DataLoch description details.
**Table S7:** University College London Hospitals description details.

## Data Availability

The data that support the findings of this study are available on request from the corresponding author. The data are not publicly available due to privacy or ethical restrictions.
