# Supplementary material for: HEalth Data Research OMOP Network (HERON‐UK), A Real‐World Evidence Federated Network of Hospital and Primary Care Electronic Health Records Including 57 Million Individuals and 7 Data Partners in the United Kingdom
Source: Pharmacoepidemiol Drug Saf. 2026 Nov 13;35(11):e70526. doi: 10.1002/pds.70526 (PMC13768135; doi:10.1002/pds.70526)
Supplement: Supplementary file 1 [file PDS-35-e70526-s001.docx]

# Supplementary material

Figure S1. CPRD Aurum geographic coverage. Person counts are provided at Integrated Care Board level.

Figure S2. Barts Health NHS Trust (Barts) geographic coverage. Person counts are provided at Lower layer Super Output Areas level.

Figure S3. Great Ormond Street Hospital NHS Foundation Trust (GOSH) geographic coverage. Person counts are provided at Integrated Care Board level. Please note the colour legend of this data partner is different from the rest.

Figure S4. Lancashire Teaching Hospitals (IDRIL) geographic coverage. Person counts are provided at Lower layer Super Output Areas level.

Figure S5. Leeds Teaching Hospitals NHS Trust (LTHT) geographic coverage. Person counts are provided at post code district level.

Figure S6. DataLoch geographic coverage. Person counts are provided at Scottish Data Zone level.

Figure S7. University College London Hospitals geographic coverage. Person counts are provided at Lower layer Super Output Areas level.

Table S1. Great Ormond Street Hospital NHS Foundation Trust description details.

*Full name:*

**Great Ormond Street Hospital NHS Foundation Trust**

*Acronym:*

GOSH

*Database description*

GOSH is a specialist tertiary care paediatrics hospital in the UK receiving children with complex and/or rare conditions. A large proportion of the children that we see typically require long-term care and follow up at GOSH. This results in data collected during routine healthcare that are very rich in number of variables and detail.

*CDM and vocabulary version:*

CDM version v5.4, Vocabulary version: v5.0 August 2024

*Time period:*

2019 to 2025

*Catchment area (Region):*

 London, South East, England, UK, and Middle East.

*Speciality:*

Paediatric hospital

*Data type:*

inpatient and outpatient data

*Data source:*

The data is sourced from Caboodle which is the data warehouse from Epic (GOSH’s EPR system).

Current observation period definition:

Start at first inpatient or outpatient admission and ends at extraction date.

*Source coding system:*

ICD10 for conditions, dm+d for drugs, OPCS4 for procedures, LONIC for some laboratory results. GOSH has a lot of GOSH-specific codes for data items relating to radiology procedures, flowsheet rows, and some data items are recorded as free text.

Tables

- Visit occurrence: Includes inpatients and outpatient admissions, and telehealth appointments for all patients who have visited GOSH within the period.
- Condition occurrence: populated. Includes patients coded diagnoses from data that populates the problem list.
- Drug exposure: partially populated. Includes medications which were administered to the patient during their physical time in hospital. Plans to extend to include home care medication in the form of medications which are ordered.
- Procedure occurrence: populated. Includes surgical and radiology procedures patients had.
- Measurement: partially populated. Data items with a standard common vocabulary are populated for E.g. laboratory tests. Further laboratory tests/ flowsheet information is added through manual mapping when required.
- Observation: populated. partially populated. Data items with a standard common vocabulary are populated for E.g. laboratory tests. Further laboratory tests/ flowsheet information is added through manual mapping when required.
- Device: No data.
- Death: populated, linked to NHS Spine records.

Table S2. Lancashire Teaching Hospitals description details.

*Full name:*

**Lancashire Teaching Hospitals**

*Acronym:*

IDRIL

*Database description*
This dataset provides hospital-based data from the Northwest of England, covering approximately 1.5 million patients. The dataset includes a wide range of clinical information, such as outpatient clinic attendances, laboratory test results (including blood tests and microbiology), and detailed cancer pathway data, including staging and grading.

*CDM and vocabulary version:*

CDM version v5.4, v5.0 30-AUG-24

*Time period:*

2005 to 2025

*Catchment area (Region):*

Lancashire

*Speciality:*

IDRIL is a major provider of secondary care and a regional and national centre for tertiary and quaternary services.

*Data type:*

Inpatient and outpatient data

*Current observation period definition:*

Individuals are in observation while they have an ongoing visit or prescription, episodes separated by 18 months or less are collapsed into a single observation episode.

Table S3. Leeds Teaching Hospitals NHS Trust description details.

*Full name:*

**Leeds Teaching Hospitals NHS Trust (LTHT)** **OMOP**

*Acronym:*

LTHT

*Database description*

This OMOP database contains routinely collected healthcare data from patients treated at Leeds Teaching Hospitals NHS Trust. It includes structured information on demographics, diagnoses, admissions, treatments, medications, laboratory results, and clinical measurements. The data have been transformed into the OMOP Common Data Model (CDM) version 5.4, enabling standardised analytics and cross-site research. The database covers a period from 2003 to 2025, with a total population of approximately 1.5 million patients.

*CDM and vocabulary version:*

CDM version v5.4, Vocabulary version: v5.0 August 2024

*Time period:*

2003 to 2025

*Catchment area (Region):*

West Yorkshire and the Humber

*Speciality:*

LTHT is a major provider of secondary care and a regional and national centre for tertiary and quaternary services. Its specialties include cancer, neurosciences, cardiac care, transplantation, major trauma, paediatrics, and genomics.

*Data type:*

Inpatient and outpatient data

*Data source:*

The data is sourced from Patient Pathway Manager (PPM – LTHT’s electronic healthcare record system), Patient Administration System (PAS), and eMeds (LTHT’s electronic prescribing and medicines administration system).

*Current observation period definition:*

Start date is the date of the first record/event available for each patient; end date is the date of the last record/event of the patient.

*Source coding system:*

ICD10 for conditions, OPCS4 for procedures, ICD-O Histology for cancer histology data

Tables

- Visit occurrence: Includes in-patient and admissions for all patients that are or have been part of the cancer pathway, as well as all in-patient admissions for all other patients. The visit_detail table has not been populated.
- Condition occurrence: populated with all diagnoses. Source data comes from PPM (cancer) and PAS (non-cancer)
- Drug exposure: populated. Includes all anti-cancer drugs (chemotherapy and immunotherapy), and some antibiotics medication (all antibiotics that are in the WHO watchlist for antimicrobial resistance and access antibiotics). Plans to extend this to all medication prescribed, but issues with non-existent source codes, so mapping needs to be done manually based on drug name.
- Procedure occurrence: populated. Includes surgical and radiotherapy procedures delivered to patients with cancer, as well as all surgical procedures delivered to all other patients.
- Measurement: populated with weight, height, TNM staging, performance status, and metastasis location.
- Observation: populated with ethnicity, IMD quintile, clinical trial participation (cancer only) and cancer histology data. Source data comes from PPM and PAS.
- Device exposure: Not populated.
- Death: Populated from ONS.

Table S4. CPRD AURUM description details.

*Full name:*

**CPRD Aurum**

*Acronym:*

*CPRD Aurum*

*Database description*

CPRD Aurum is a database of anonymised primary care electronic health records (EHR) collected from participating general practitioner (GP) practices in England which use the EMIS® patient management software system.  CPRD Aurum captures coded information on:

CPRD receives a full historic collection of electronic health records from contributing practices, including data on deceased patients and those who have left the practice. Data is considered to be of acceptable quality for research from 1995, following the widespread adoption of GP computer systems in the UK. Around 98% of the England population is registered with a single GP practice at any time, but 5-6% of registered patients opt out of the use of their data for research. CPRD Aurum has shown to be broadly representative of the general population in England.

*CDM and vocabulary version:*

CDM Version:  v5.4. Vocabulary version: v5.0 Aug 2024

*Time period:*

1995-2024

*Catchment area (Region):*

England

*Speciality:*

Primary care

*Data type:*

GP records

*Data source:*

EMIS® patient management software system 
Clinical information including diagnoses, symptoms, results of clinical investigations and lab tests, screening and prevention activity, and referrals to specialist services. This information is captured using a combination of Read V2, SNOMED-CT UK extension, and proprietary codes.

Prescriptions for medicines and devices, captured using SNOMED CT UK Drug extension (based on the NHS Dictionary of Medicines and Devices, dm+d) and some proprietary codes.

*Current observation period definition:*

Patient observation period starts from the later of 01/01/1995 and date of registration with the practice, and ends at the date of deregistration, death, or date of last data collection from the practice, whichever is later.

*Source coding system:*

Read and snomed for conditions, dm+d for drugs, snomed for procedures, dm+d for device, observations snomed. Measurement snomed

Tables

- Condition occurrence: populated. Source data comes from EMIS Web primary care electronic health record system.
- Drug exposure: populated. Source data comes from EMIS Web primary care electronic health record system.
- Procedure occurrence: populated. Source data comes from EMIS Web primary care electronic health record system.
- Measurement: populated. Source data comes from EMIS Web primary care electronic health record system.
- Observation: populated. Source data comes from EMIS Web primary care electronic health record system.
- Device: populated. Source data comes from EMIS Web primary care electronic health record system.
- Death: populated. Source data comes from EMIS Web primary care electronic health record system.
- Visit occurrence: populated. Source data comes from EMIS Web primary care electronic health record system.

Table S5. Barts Health NHS Trust description details.

*Full name:*

**Barts Health NHS Trust**

*Acronym:*

*Barts*

*Database description*

Barts Health NHS Trust is the largest NHS hospital group or Trust in the UK, comprising six hospitals; The Royal London Hospital, Newham General Hospital, Whipps Cross Hospital, Mile End Hospital, St Bartholomew’s Hospital and the London NHS Nightingale Hospital, a purpose-built COVID-19 hospital.

*CDM and vocabulary version:*

Cdm version : v5.4, vocabulary version v5.0 30-AUG-24

*Time period:*

2012 – 2025 (although records begin properly from 2015/2016 due to data migration and onboarding of e-prescribing and sites joining).

*Catchment area (Region):*

The hospitals serve the ethnically diverse communities of over 3.2 million people in East London. Local primary care provision for this population is organised within five local boroughs - the City of London, Hackney, Newham, Tower Hamlets and Waltham Forest.

*Speciality:*

Secondary and tertiary care provider, including Level 1 major trauma centre, specialist cardiac, and cancer services, neurosurgery and paediatrics

*Data type:*

inpatient and outpatient data, drug and device data

*Data source:*

The data source is a single co-ordinated data warehouse.  This takes data from many sources shown on the schema diagram (attached). The principal data flow is from the Oracle database manged by Cerner. The Barts Health deployment of Cerner Millennium is at the enterprise level and is available at all our sites and remotely using VDI connections. The main EHR component module is called Powerchart, and we currently run on 2015 code but are currently upgrading the codebase to 2018 code.

Other data sources into Millennium are feeder systems that include radiology (Sectra), pathology (Clinsys Winpath) and cancer chemotherapy (Aria)

*Current observation period definition:*

Min – maximum from first appointment/ entry to latest.

*Source coding system:*

ICD10 and SNOMED for conditions, OPCS codes for procedures

Tables

- Visit occurrence: populated. Source data comes from Cerner Millennium EHR
- Condition occurrence: populated. Source data comes from Cerner Millennium EHR
- Drug exposure: populated. Source data comes from Cerner Millennium EHR
- Procedure occurrence: populated. Source data comes from Cerner Millennium EHR
- Measurement: populated. Source data comes Cerner Millennium EHR
- Observation: populated. Source data comes from Cerner Millennium EHR
- Device: populated. Source data comes Cerner Millennium EHR
- Death: populated, linked to ONS

Table S6. DataLoch description details.

*Full name:*

DataLoch – Edinburgh Cancer Informatics

*Acronym:*

DataLoch

*Database description*

The Dataloch OMOP data currently includes secondary care data from NHS Lothian data. Serving a population of 1.4 million, the Edinburgh Cancer Centre has for 40 years maintained a clinical process and outcomes database that integrates with regional electronic health systems and describes the complete cancer journey.

*CDM and vocabulary version:*

CDM version v5.4, Vocabulary version: v5.0 October 2021

*Time period:*

1996 to 2021

*Catchment area (Region):*

NHS Lothian (South-East of Scotland)

*Speciality:*

Oncology

*Data type:*

Diagnosis, treatment, follow up, inpatient, outpatient

*Data source:*

The current mapped data (May 2025) includes the cancer registry (SMR06), inpatient data (SMR01), outpatient data (SMR00), and community prescribing (PIS). More info here: <https://cancer-data.ecrc.ed.ac.uk/about/the-data/>. Additional mapping is expected soon for Chemotherapy.

Current observation period definition:

Earliest record as start date, and death date or date of extraction as end date

*Source coding system:*

dm+d for drugs, snomed anf hcpcs for measurements, icd10 conditions, opcs4 for procedures.

Tables

- Visit occurrence: populated. Source data comes from SMR01
- Condition occurrence: populated. Source data comes from SMR01 (that is because for this study we are using a modified version of the Covid ETL)
- Drug Exposure: populated. Source data comes from PIS
- Procedure Occurrence: populated. Source data comes from SMR01
- Measurement: populated: Source data comes from NHS Lothian lab data from TRAK and Lighthouse Lab data (Covid tests)
- Observation: populated: Some of our conditions data is mapped to Observation because the ICD10 source code maps to a standard code in the Observation domain. The table also contains our SIMD mappings.
- Device: not populated
- Death: Populated: Source data comes from NRS.

Table S7. University College London Hospitals description details.

*Full name:*

**University College London Hospitals NHS Foundation Trust**

*Acronym:*

**UCLH**

*Database description*

This UCLH database contains standardised healthcare data in OMOP CDM.

It includes inpatient and outpatient data from 2019 to 2025 with a total population of about 1.4 million patients.

Epic EHR data on data on demographics, admissions, diagnosis, medications, laboratory results and procedures were translated into OMOP format to support observational research and analytics.

*CDM and vocabulary version:*

CDM version v5.4, Vocabulary version: v5.0 27-FEB-25

*Time period:*

2019 to 2025

*Catchment area (Region):*

London

*Speciality:*

UCLH is one of the largest NHS trusts in the United Kingdom and provides first-class acute and specialist services such as critical care, cancer, haematology, and general surgery.

*Data type:*

Inpatient and outpatient data

*Data source:*

The data source is Epic's database Caboodle.

Current observation period definition:

First visit start date to the earlier of last visit end date or date of death.

*Source coding system:*

RxNorm and dm+d for drugs, dm+d for medications in device exposure, LOINC for laboratory result, SNOMED for conditions, specimen and observation, SNOMED and OPCS4 for procedures

Tables

- Visit occurrence: populated. Includes inpatients and outpatient admisions. Source data comes from Epic EHR.
- Condition occurrence: populated. Includes patients coded diagnosis from data that populates problem list and billing diagnosis. Source data comes from Epic EHR.
- Drug exposure: populated. Includes administered drugs to inpatients and prescribed or ordered drugs to outpatients. Source data comes from Epic EHR.
- Procedure occurrence: populated. Includes surgical, imaging and procedures from flowsheets. Source data comes from Epic EHR.
- Measurement: populated. Includes mainly laboratory results. Source data comes from Epic EHR.
- Observation: populated. Includes mainly data from flowsheets. Source data comes from Epic EHR.
- Device: populated. Includes data from flowsheets and administered drugs for inpatients and outpatients. Source data comes from Epic EHR.
- Death: populated. Source data comes from Epic EHR.
